# Integrated Transcriptomic and Metabolomic Analysis Reveals the Regulation Network of CEBiP in Rice Defense Against *Magnaporthe oryzae*

**DOI:** 10.3390/ijms26115194

**Published:** 2025-05-28

**Authors:** Qi Zheng, Jiandong Bao, Lin Li, Zifang Shen, Jiaoyu Wang, Asen Daskalov, Xueming Zhu, Fucheng Lin

**Affiliations:** 1 State Key Laboratory for Quality and Safety of Agro-Products, Zhejiang Provincial Key Laboratory of Agricultural Microbiomics, Key Laboratory of Agricultural Microbiome (MARA), Institute of Plant Protection and Microbiology, Zhejiang Academy of Agricultural Sciences, Hangzhou 310021, China; qi.zheng@zaas.ac.cn (Q.Z.); baojd@zaas.ac.cn (J.B.); lilin718@zaas.ac.cn (L.L.); zfshen@zju.edu.cn (Z.S.); wangjiaoyu78@sina.com (J.W.); asen.daskalov@u-bordeaux.fr (A.D.); 2 Xianghu Laboratory, Hangzhou, 311231, China; 3 ImmunoConcEpT, CNRS UMR 5164, University of Bordeaux, 33076 Bordeaux, France

**Keywords:** *Magnaporthe oryzae*, rice blast disease, CEBiP, pattern recognition receptor, transcriptomics, metabolomics

## Abstract

Rice blast disease is a major threat to rice yields. Sustainable control relies on resistant varieties, where plant immunity is triggered by pattern recognition receptors like receptor-like proteins (RLPs). The rice RLP chitin-elicitor binding protin (CEBiP) recognizes fungal chitin and confers blast resistance to pathogen *Magnaporthe oryzae*. However, understanding of the broader signaling and metabolomic pathways associated with CEBiP activation remains limited. Here, we performed an integrated transcriptomic and metabolomic analysis of the rice Zhonghua 11 genotype and CEBiP knockout plants. Both plants were infected with *M. oryzae*, and infected leaves were harvested at 24, 48, and 72 hpi for RNA sequencing and Liquid Chromatography-Tandem Mass Spectrometry analysis. Transcriptomics identified a total of 655 genes that were differentially regulated upon knockout of CEBiP; they were mainly related to diterpenoid/phenylpropanoid biosynthesis, nitrogen metabolism, the mitogen-activated protein kinasesignaling pathway, plant–pathogen interaction, and plant hormone signal transduction. The presence of a large number of pathogenesis-related protein 1 family genes indicates the key role of salicylic acid (SA) in CEBiP immunity. Metabolomics detected a total of 962 differentially accumulated metabolites and highlights the roles of caffeine and glutathione metabolism in CEBiP-mediated immunity. Since caffeine and glutathione metabolism can regulate SA signaling, we propose that SA signaling plays a central role in the CEBiP immune function.

## 1. Introduction

Rice blast disease is one of the most damaging plant diseases and causes an estimated annual yield loss of 10–30% of rice production globally [1]. The disease is caused by the fungal pathogen *Magnaporthe oryzae*, which has diverged from *Magnaporthe grisea*, based on phylogenetic analyses and uncovered reproductive isolation [2]. Historically, rice blast has been managed primarily through the application of fungicides. However, this strategy poses environmental concerns, while the overuse of fungicides results in the selection of fungicide-resistant *M. oryzae* strains [3]. An alternative strategy involves the deployment of rice cultivars with genetic resistance to *M. oryzae*. Nevertheless, resistance-breaking pathogen populations can rapidly arise through genetic mutations and adaptive evolution [4]. To address these challenges, a large number of researchers are focusing on fundamental questions in regard to the interaction between rice (*Oryza sativa*) and *M. oryzae*, studying it at the molecular level. Over recent decades, significant progress has been made in elucidating the mechanisms underlying pathogen recognition and the activation of plant immune responses.

Plant cell surface-located pattern recognition receptors (PRRs) can detect pathogen-associated molecular patterns (PAMPs) or damage-associated molecular patterns (DAMPs) to initiate an immune response, known as pattern-triggered immunity (PTI) [5,6]. PTI response is associated with a series of reactive oxygen species (ROS) production, mitogen-associated protein kinases (MAPKs) activation, and sometimes hypersensitive response (HR) [7,8,9]. PRRs consist of receptor-like kinases (RLKs) and receptor-like proteins (RLPs) [7,8,9]. RLKs contain an extracellular domain, a transmembrane domain, and an intracellular kinase domain. RLPs have an overall similar structure to RLKs but lack an intracellular kinase domain [7,8,9]. The extracellular domain is responsible for recognizing PAMPs/DAMPs, such as bacterial flagellin and fungal chitin [10,11]. The intracellular kinase domain of RLKs is essential for signal transduction inside the plant cells [12,13]. Since RLPs lack the intracellular kinase domain, they recruit one or multiple RLKs and form a receptor complex to initiate downstream signaling [12,13]. To date, several rice PRRs have been identified to be involved in immunity against *M. oryzae* infection [14,15].

The rice RLP CEBiP is a chitin oligosaccharide elicitor-binding protein [16]. Chitin is an important component of the fungal cell wall and is recognized as a typical PAMP, inducing plant defense responses [17,18]. CEBiP mediates immune response upon the treatment of chitin oligosaccharide N-acetylglucosamine (GlcNAc)_8_, and the knockdown of CEBiP impairs the chitin elicitor-induced immune response in rice cells [16]. Knockdown of CEBiP in rice also results in enhanced invasion of *M. oryzae* [19]. Due to the lack of an intracellular domain, CEBiP cooperates with rice RLK Chitin Elicitor Receptor Kinase (CERK) to activate downstream immune signaling [20]. Although CERK is essential for chitin elicitor-induced signals in rice [11,20], it cannot directly bind to chitin [20]. Upon chitin binding, CEBiP recruits the co-receptor CERK to form a heterodimer at the plasma membrane in rice cells [20]. In response to the chitin elicitor, CEBiP/CERK mediates a series of immune responses in rice cells, including MAPK activation, reactive oxygen species burst, and gene expression changes [11,16,20]. However, the global network of gene expression and associated metabolites production downstream of CEBiP-mediated immunity against pathogen *M. oryzae* infection remains poorly characterized.

In this study, we aim to uncover hallmarks of CEBiP-mediated rice immunity triggered by *M. oryzae* at the early stages of infection. We infected the rice Zhonghua11 (ZH11) genotype and CEBiP knockout mutant plants with *M. oryzae* and harvested leaves at 24, 48, and 72 h post infection (hpi) for RNA sequencing and LC-MS/MS analysis. We identified a total of 655 genes and 962 metabolites that were specifically differentially regulated in response to the knockout of CEBiP. The transcriptomics analysis highlights the importance of pathogenesis-related protein 1 family genes in response to CEBiP-mediated signaling. The metabolomic analysis suggests the essential role of caffeine metabolism and glutathion metabolism. Integrated transcriptomic and metabolomic analysis indicates the importance of salicylic acid signaling in the CEBiP-mediated defense response against *M. oryzae*. Eventually, we attempt to integrate our findings into the existing paradigm of plant immunity.

## 2. Results

### 2.1. Knockout of the CEBiP Led to a Mild Increase in the Severity of Disease Symptoms upon M. oryzae Infection

**Figure 1:**
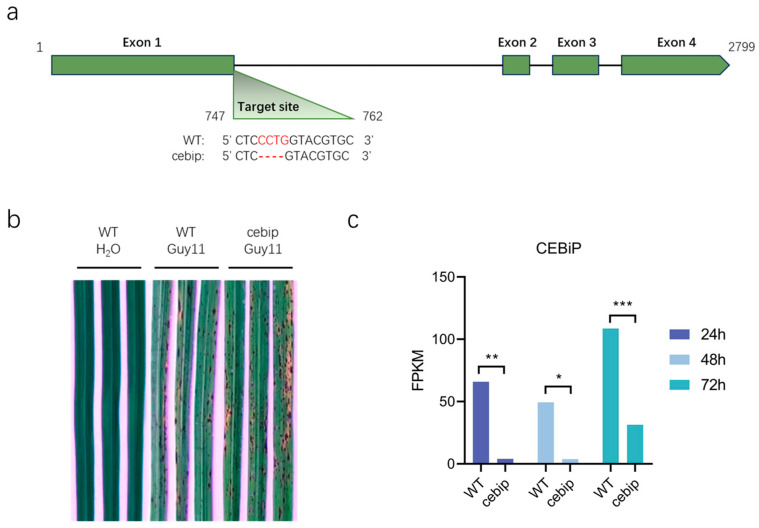
Knockout of CEBiP leads to a mild increase in disease symptoms in rice leaves. (**a**) CRISPR-Cas9-mediated mutation of CEBiP in the rice Zhonghua 11 (ZH11) line. The green boxes represent exons. The targeted sequence of CEBiP is shown in red. (**b**) Disease symptoms of the wild-type (WT) and *cebip* lines infected by the *Magnaporthe oryzae* Guy11 strain. Images were taken at 5 days post inoculation. (**c**) Transcriptional expression of CEBiP in the WT and *cebip* lines infected with the *M. oryzae* Guy11 strain. Data were analyzed from the RNAseq sequencing results. The bar represents the mean transcript level of CEBiP in three biological replicates of each treatment. The significance of differences is indicated by “*”. “*” represents *p* < 0.05, “**” represents *p* < 0.01, and “***” represents *p* < 0.001.

To investigate the global gene network and associated metabolites that specifically respond to the CEBiP-mediated defense response to *M. oryzae* infection, we first obtained the CEBiP knockout mutant line (*cebip*, from here onwards) via CRISPR-Cas9-mediated gene editing by using the rice Zhonghua 11 (ZH11) genotype. Sanger sequencing confirmed a mutation at the last four bases of exon 1 in the *cebip* line (Figure 1a). Next, we infected the *cebip* and ZH11 wild type (WT) lines with the *M. oryzae* Guy11 strain and examined the disease symptoms. The WT line inoculated with H_2_O served as a negative control, and as expected, showed no disease symptoms (Figure 1b). The WT line infected with *M. oryzae* exhibited typical blast, such as yellow leaves and spot lesions, whereas the *cebip* line infected with *M. oryzae* displayed slightly stronger disease symptoms. These results suggest that CEBiP contributes positively to rice resistance against *M. oryzae*.

In this study, we focused on the role of CEBiP in rice immunity during the early stages of *M. oryzae* infection. Therefore, we inoculated the WT and *cebip* lines with the *M. oryzae* Guy11 strain and harvested the rice leaves at 24 hpi, 48 hpi, and 72 hpi, prior to the appearance of visible disease symptoms. The leave samples were collected for subsequent transcriptomic and metabolomic analysis. To confirm the knockout of CEBiP in the *cebip* line, we analyzed the transcript level of CEBiP (Os03g0133400) from the transcriptome data. Compared to the *cebip* line, CEBiP showed significantly higher expression in the WT line, particularly at 72 hpi (Figure 1c). In the *cebip* line, CEBiP expression remained very low (<10 FPKM) at 24–48 hpi but increased sharply at 72 hpi, though it was still substantially lower than in WT at the same time point (Figure 1c). Thus, we confirmed the differences in the expression level of CEBiP between the WT and *cebip* lines.

### 2.2. Transcriptome Analysis Reveals a Set of Differentially Expressed Genes Involved in CEBiP Immune Function

For the transcriptome study, we collected three biological replicates for both the WT and *cebip* groups at 24 hpi, 48 hpi, and 72 hpi, resulting in a total of 18 samples for RNA sequencing. After quality filtering, each sample obtained clean data, ranging from 5.65 to 7.01Gb, with the Q30 value over 94.83% (Supplemental Table S1); these data were used for downstream analysis. To investigate which factors influence the variance in the mRNA abundance in our samples, we performed principal component analysis (PCA). The major PC1 was 23.43% and was linked to time points, and the second PC2 was 16.42% and was associated with the plant genotype (Supplemental Figure S1). Together, time and the plant genotype were key contributors to the observed transcriptional variation.

**Figure 2:**
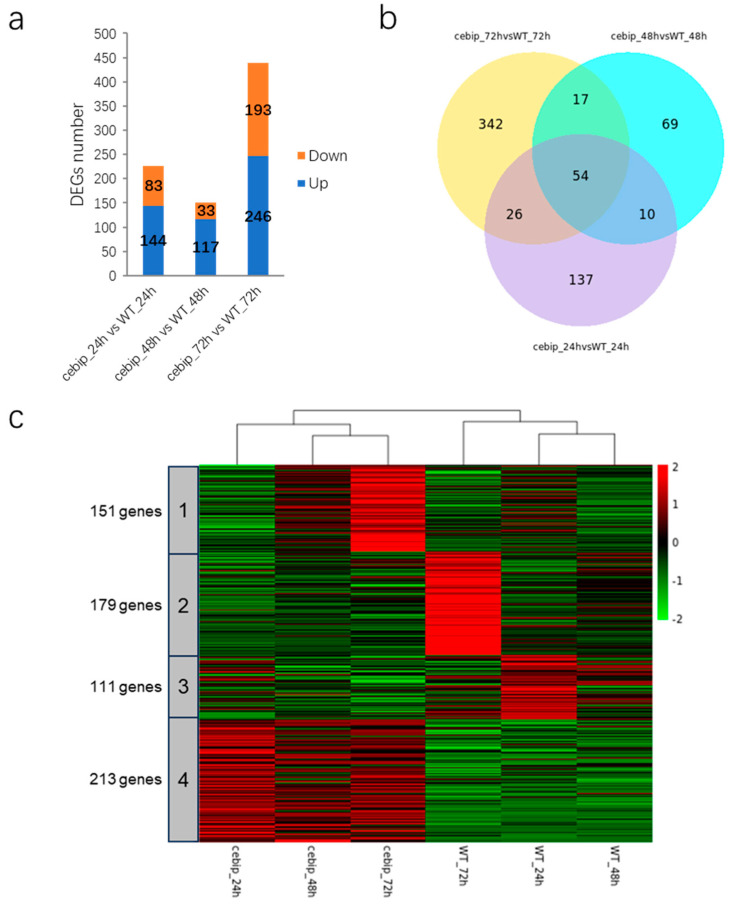
DEGs analysis between the *cebip* vs. WT groups. (**a**) Histogram of the number of significantly up- and down-regulated DEGs (padj < 0.05, log_2_FC > 2) between the *cebip* vs. WT group at 24 hpi, 48 hpi, and 72 hpi. (**b**) Venn diagram of significantly differentially expressed DEGs between the *cebip* vs. WT groups. In total, 655 genes were identified as significantly expressed DEGs between the *cebip* vs. WT groups. (**c**) K-means clustering of 655 DEGs between the *cebip* vs. WT groups.

To investigate which genes are affected by plant genotypes, which represent the absence or presence of the CEBiP gene in the genome background, we compared the transcriptional expression level of genes between the *cebip* vs. WT groups. The number of differentially expressed genes (DEGs) increased over time, with the most pronounced changes observed at 72 hpi (Figure 2a). Comparing the *cebip* vs. WT groups, there were 227 (144 up-regulated DEGs and 83 down-regulated DEGs), 150 (117 up-regulated DEGs and 33 down-regulated DEGs), and 439 (246 up-regulated DEGs and 193 down-regulated DEGs) DEGs showing significantly differential regulation at 24 hpi, 48 hpi, and 72 hpi (padj < 0.05, log2FC > 2), respectively. To visualize the overlap of these DEGs among different treatments, we performed a Venn diagram analysis. This revealed 655 DEGs affected by both the plant genotype and time points (Figure 2b). Among them, 137, 69, and 342 DEGs were uniquely expressed at 24 hpi, 48 hpi, and 72 hpi, respectively. Overall, 10 DEGs were differentially expressed at both 24 hpi and 48 hpi, 17 between 48 and 72 hpi, and 26 between 24 and 72 hpi. Only 54 DEGs showed differential expression at all time points. Next, to visualize the expression pattern of the 655 DEGs, we performed a K-means clustering analysis for all the groups. The clustering revealed that the plant genotype was the main factor shaping DEG profiles, and these were categorized into four distinct clusters (Figure 2c). Cluster 1 included 151 DEGs with the highest expression level in the *cebip* groups at 72 hpi, while cluster 2 contained 179 DEGs displaying the highest expression level in the WT groups at the same time point. In total, 330 DEGs were specifically expressed at 72 hpi, accounting for half of the total 655 DEGs, and are likely involved in downstream signaling activation. Cluster 3 contained 111 DEGs showing a relatively higher expression level in the WT groups at 24 hpi, and these DEGs may play a key role in pathogen recognition. Cluster 4 comprised 213 DEGs presenting an overall higher expression level in the *cebip* groups at all time points, and they were specifically regulated in response to the absence of CEBiP.

### 2.3. GO and KEGG Enrichment Analysis of Significantly Differentially Expressed Genes

**Figure 3:**
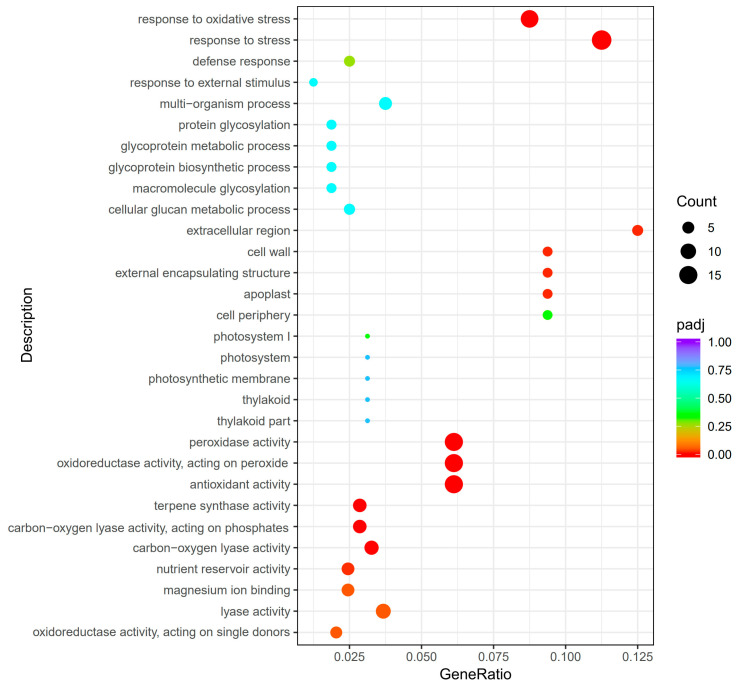
Scatterplot of the GO enrichment analysis of 655 significantly differentially expressed genes in the *cebip* vs. WT groups.

To understand the function of those significantly differentially expressed genes, we performed the Gene Ontology (GO) enrichment analysis for the above 655 DEGs. The significantly enriched pathways (padj < 0.05) involved in biological processes include response to oxidative stress and response to stress (Figure 3). The cellular component analysis indicates that these DEGs were dominantly located in the compartment surrounding the cell membrane, like the cell wall, extracellular region, external encapsulating structure, and apoplast (Figure 3). As for the molecular functions, these DEGs were significantly associated with peroxidase activity, antioxidant activity, terpene synthase activity, carbon–oxygen lyase activity, nutrient reservoir activity, magnesium ion binding, lyase activity, oxidoreductase activity, and dioxygenase activity (Figure 3).

**Figure 4:**
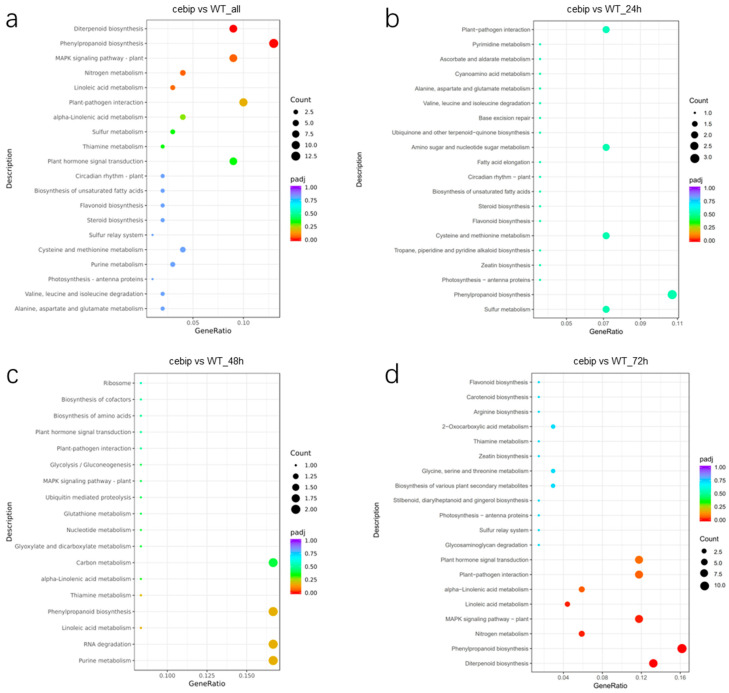
Scatterplot of the KEGG enrichment analysis of significantly differentially expressed genes between the *cebip* vs. WT groups (**a**) for all 655 DEGs and at (**b**) 24 hpi, (**c**) 48 hpi, and (**d**) 72 hpi.

To understand the downstream pathways implicated in CEBiP regulation, we performed the Kyoto Encyclopedia of Genes and Genomes (KEGG) enrichment analysis for 655 DEGs. The result shows that these DEGs are significantly enriched (padj < 0.05) in diterpenoid biosynthesis (9 DEGs) and phenylpropanoid biosynthesis (13 DEGs) (Figure 4a). Meanwhile, a large number of DEGs were associated with plant defense-related categories like MAPK signaling pathway (9 DEGs), plant–pathogen interaction (10 DEGs), and plant hormone signal transduction (9 DEGs).

We also performed KEGG analysis for DEGs differentially expressed at the individual time point, as the k-means clustering analysis suggests that a large number of DEGs show remarkable expression at 24 hpi and 72 hpi (Figure 2c). At 24 hpi, the DEGs were mainly involved in plant–pathogen interaction (two DEGs), amino sugar and nucleotide sugar metabolism (two DEGs), cysteine and methionine metabolism (two DEGs), phenylpropanoid biosynthesis (three DEGs), and sulfur metabolism (two DEGs) (Figure 4b). The DEGs showing differential expression at 48 hpi were mainly associated with carbon metabolism (two DEGs), phenylpropanoid biosynthesis (two DEGs), RNA degradation (two DEGs), and purine metabolism (two DEGs) (Figure 4c). However, none of the above pathways were significantly enriched at either 24 hpi or 48 hpi. Interestingly, the KEGG pathways enriched at 72 hpi exhibited a substantial overlap with those identified from all 655 DEGs (Figure 4d). The significantly enriched pathways included diterpenoid biosynthesis (9 DEGs), phenylpropanoid biosynthesis (11 DEGs), nitrogen metabolism (4 DEGs), MAPK signaling pathway–plant (8 DEGs), and linoleic acid metabolism (3 DEGs) (Figure 4d). In addition, alpha-linolenic acid metabolism (four DEGs), plant–pathogen interaction (eight DEGs), and plant hormone signal transduction (eight DEGs) were also highly enriched. These findings provide strong evidence that DEGs differentially regulated at 72 hpi are closely linked to CEBiP-mediated downstream signaling.

Next, we closely examined specific individual DEGs from defense-related categories, including plant–pathogen interaction, MAPK signaling pathway, and plant hormone signal transduction. The plant–pathogen interaction (dosa04626) category includes the genes encoding a chitin elicitor-binding protein CEBiP, a series of pathogenesis-related (PR) proteins, a mitogen-activated protein kinase protein (MAPK5), a 3-ketoacyl-CoA synthase 6, and an unknown protein (Table 1). The MAPK signaling pathway (dosa04016) and the plant hormone signal transduction (dosa04075) categories share the same genes, which encode PR family proteins, a MAPK5 protein, an ethylene response sensor ERS2, and a protein phosphatase PP2C1. Notably, PR proteins and MAPK5 were also enriched in the plant–pathogen interaction category, indicating an essential role of these proteins in CEBiP-mediated downstream signaling.

### 2.4. Defense-Related DEGs Analysis

**Figure 5:**
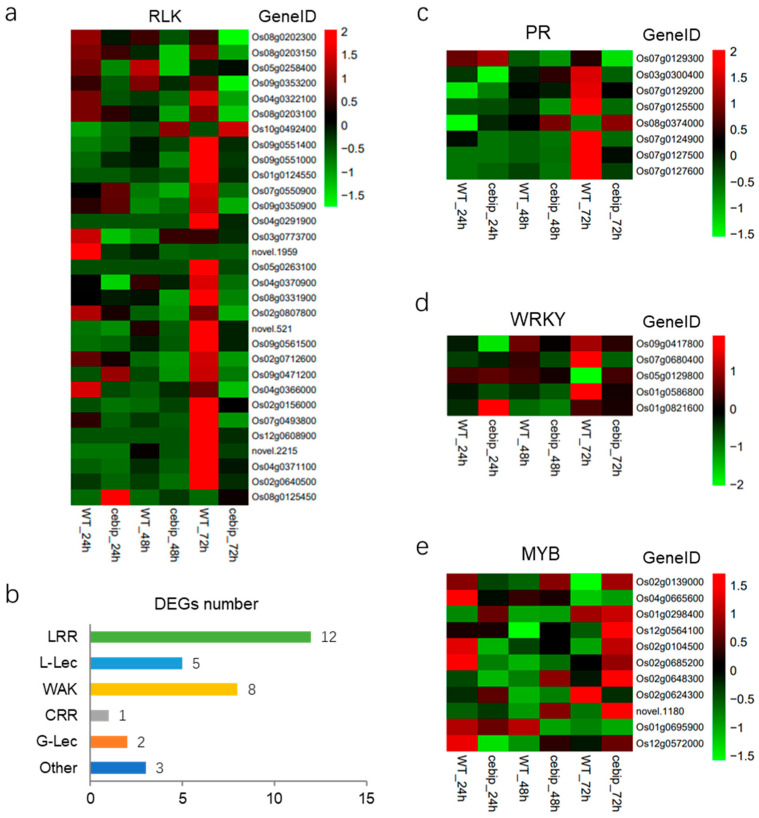
Heat maps showing the expression patterns of defense-related genes that were identified with significantly differential expression between the *cebip* vs. WT groups. (**a**) Expression pattern of RLK DEGs. (**b**) Categories of RLK DEGs. (**c**) Expression pattern of PR DEGs. (**d**) Expression pattern of WRKY DEGs. (**e**) Expression pattern of MYB DEGs.

Receptor-like protein kinases (RLKs) are important for the function of receptor-like proteins (RLPs), which lack an intracellular kinase domain and depend on RLKs for signal transduction [7,13]. To explore how RLKs are involved in the CEBiP-mediated defense response against *M. oryzae*, we analyzed the expression level of RLKs from 655 significantly differentially expressed genes. In total, 31 DEGs encoding RLK proteins were significantly expressed upon knockout of CEBiP (Figure 5a). The RLK class was composed of twelve DEGs encoding LRR receptor-like serine/threonine-protein kinase, two DEGs encoding G-type lectin (G-Lec) S-receptor-like serine/threonine-protein kinase, eight DEGs encoding wall-associated (WAK) receptor kinase, five DEGs encoding L-type lectin (L-Lec)-domain containing receptor kinase, one DEG encoding cysteine-rich receptor-like (CRR) protein kinase, and three DEGs encoding the other type of RLK (Figure 5b).

Most RLK genes (29 out of 31) displayed an overall higher expression pattern in the WT groups (Figure 5a). Specifically, sixteen DEGs were particularly highly induced at 72 hpi, and two DEGs (Os03g0773700 and novel.1959) were highly expressed at 24 hpi. Five DEGs were initially induced at 24 hpi, decreased at 48 hpi, and then increased again at 72 hpi. The other six DEGs showed an average expression level among different time points. In addition, two DEGs (Os10g0492400 and Os08g0125450) present a generally higher expression pattern in the *cebip* groups (Figure 5a). The DEG (Os10g0492400) exhibited a higher expression level in the *cebip* group, and the expression level increased as time developed. Another DEG (Os08g0125450) was specifically induced in *cebip* groups at 24 hpi. We also investigated the relationship between the expression patterns and types of RLK DEGs, but no significant correlation was observed. Overall, most RLK and RK DEGs were negatively regulated upon knockout of CEBiP, suggesting a positive function of them in the OsCEBiP-mediated defense response.

Pathogenesis-related (PR) genes play a crucial role in plant immunity against pathogen infection [21,22]. There were eight DEGs encoding PR protein significantly expressed upon knockout of CEBiP (Figure 5c). Six of them (Os07g0129300, Os07g0129200, Os07g0125500, Os07g0124900, Os07g0127500, and Os07g0127600) belong to the PR1 family, and another two (Os03g0300400 and Os08g0374000) are from the PR10 family. The majority of PR DEGs were specifically induced in the WT group at 72 hpi (Figure 5c). PR1 (Os07g0129300) had relatively higher expression at 24 hpi in both the WT and *cebip* groups. BetvI (Os08g0374000) presented a higher expression level in the *cebip* group, and the expression level decreased as time developed. These data suggest that PR1 and PR10 family proteins are highly involved in CEBiP-mediated defense signaling.

Transcription factor (TF) genes are essential for transcriptional reprogramming in the nucleus upon plant immune activation [23,24,25]. We noticed five WRKY DEGs and eleven MYB DEGs present significant expression in the absence of CEBiP (Figure 5d,e). For WRKY family DEGs, WRKY62 (Os09g0417800), WRKY47 (Os07g0680400), and WRKY27 (Os01g0586800) were generally down-regulated in the absence of CEBiP (Figure 5d). WRKY109 (Os05g0129800) notably dropped in the WT group at 72 hpi, while WRKY21 (Os01g0821600) was specifically highly expressed in the *cebip* group at 24 hpi. As for MYB family DEGs, eight MYB DEGs (Os02g0139000, Os01g0298400, Os12g0564100, Os02g0104500, Os02g0685200, Os02g0648300, novel.118, and Os12g0572000) increased their expression level in the *cebip* group as time developed (Figure 5e). Six MYB DEGs (Os02g0139000, Os04g0665600, Os02g0104500, Os02g0685200, Os01g0695900, and Os12g0572000) showed the decreased expression pattern in the WT group as time developed. Generally, WRKY and MYB DEGs are highly involved in the CEBiP-mediated response against *M. oryzae*.

### 2.5. Differentially Accumulated Metabolites Analysis Reveals a Set of Metabolites Involved in CEBiP-Mediated Defense Response

**Figure 6:**
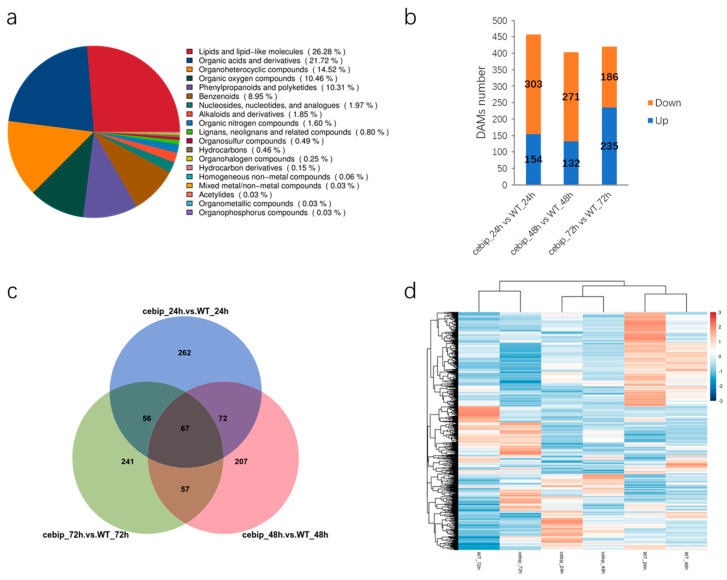
Differentially accumulated metabolites analysis between the *cebip* vs. WT groups. (**a**) Classification of all detected metabolites. (**b**) Number of DAMs significantly accumulated at 24 hpi, 48 hpi, and 72 hpi. *p* value <0.5, log_2_FC >1.5, VIP > 1. (**c**) Venn diagram to show the overlap of DAMs among the 3 time points. (**d**) Heatmap to show accumulation patterns of DAMs among all treatments.

To investigate the metabolites and metabolic pathways involved in the CEBiP function, we conducted the same *M. oryzae* infection assay as in the transcriptomic study in both the WT and *cebip* lines and collected rice leave samples for metabolomic analysis. In total, 3304 metabolites were obtained and divided into 19 categories (Figure 6a). The major categories of compounds identified included lipids and lipid-like molecules (26.28%), organic acids and derivatives (21.72%), organoheterocyclic compounds (14.52%), organic oxygen compounds (10.46%), phenylpropanoids and polyketides (10.31%), and benzenoids (8.95%), while the remaining categories each accounted for less than 2% of the total (Figure 6a). The number of the significantly differentially accumulated metabolites (DAMs) (*p* value < 0.5, log2FC > 1.5, VIP > 1) between the *cebip* vs. WT group reached 457, 403, and 421 at 24 hpi, 48 hpi, and 72 hpi, respectively (Figure 6b). In detail, 457 DAMs from 24 hpi contained 154 DAMs that were up-regulated and 303 DAMs that were down-regulated. For DAMs from 48 hpi, 132 out of 403 DAMs showed up-regulation, and the remaining 271 DAMs showed down-regulation. As for 421 DAMs from 72 hpi, 235 DAMs presented up-regulation, and the other 186 DAMs presented down-regulation.

To visualize the overlap of the above DAMs among different time points, we performed the Venn diagram analysis. The result shows that there were 262, 207, and 241 DAMs uniquely accumulated at 24 hpi, 48 hpi, and 72 hpi, respectively (Figure 6c). In total, 72 DAMs were specifically accumulated at both 24 and 48 hpi, 57 DAMs were specifically detected at both 48 and 72 hpi, and 56 DAMs were specifically accumulated at both 24 and 72 hpi. Meanwhile, 67 DAMs were commonly detected at all three time points. In total, 962 DAMs were significantly expressed in the absence of OsCEBiP, and they were used for further analysis. Next, we examined the accumulation pattern of these DAMs among all treatments. Heatmap analysis showed that DAMs accumulated at 72 hpi were different from those that accumulated at 24–48 hpi, and DAMs were closely clustered based on the plant genotypes during 24–48 hpi (Figure 6d).

### 2.6. KEGG Enrichment Analysis of Significantly Expressed DAMs

**Figure 7:**
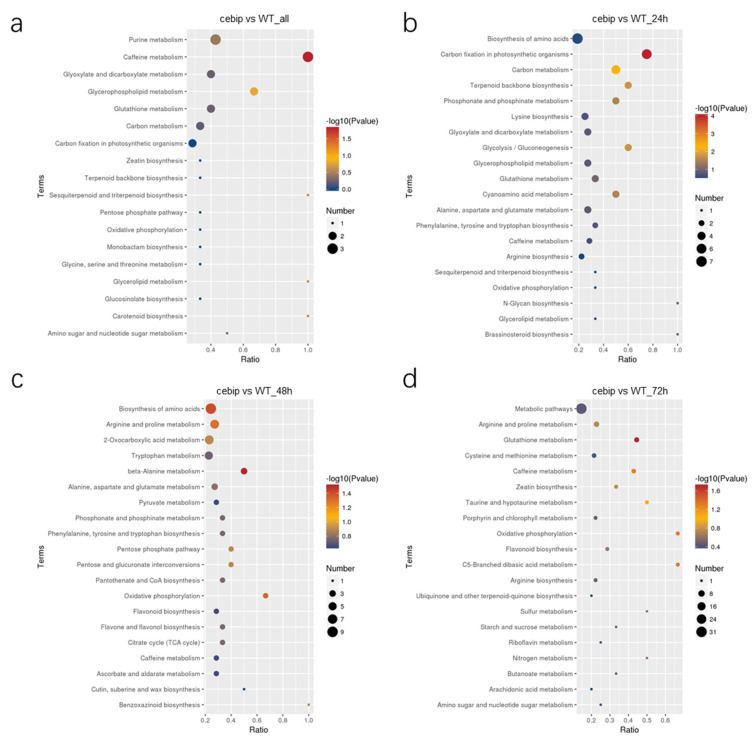
KEGG analysis of DAMs in between the *cebip* vs. WT groups for (**a**) all DAMs and at (**b**) 24 hpi, (**c**) 48 hpi, and (**d**) 72 hpi.

To investigate the function of DAMs that are involved in the CEBiP-mediated defense response, we performed a KEGG enrichment analysis for all 962 DAMs, as well as the DAMs differentially accumulated at the individual time points. The only significantly enriched pathway for all 962 DAMs is the caffeine metabolism pathway (*p* value < 0.05), which consisted of 3 DAMs (Figure 7a). As for the individual time point, there were six pathways significantly enriched at 24 hpi: carbon fixation in photosynthetic organisms (six DAMs), carbon metabolism (five DAMs), glycolysis/gluconeogenesis (three DAMs), terpenoid backbone biosynthesis (three DAMs), phosphonate and phosphinate metabolism (three DAMs), and cyanoamino acid metabolism (three DAMs) (Figure 7b). DAMs accumulated at 48 hpi were mainly enriched in beta-alanine metabolism (three DAMs), biosynthesis of amino acids (nine DAMs), and oxidative phosphorylation (two DAMs) (Figure 7c). The significantly enriched pathway at 72 hpi includes glutathione metabolism (four DAMs), oxidative phosphorylation (two DAMs), C5-Branched dibasic acid metabolism (two DAMs), and caffeine metabolism (three DAMs) (Figure 7d). Overall, during 24–48 hpi, most metabolites were associated with the biosynthesis process, whereas at 72 hpi, large metabolites were associated with metabolic activity. Notably, glutathione metabolism and caffeine metabolism were frequently detected in the top 20 KEGG pathways, whether analyzing the complete set of 962 DAMs or examining DAMs at individual time points, suggesting a pivotal role of these two metabolisms in CEBiP immune function.

### 2.7. Correlation Analysis Between Transcriptomic and Metabonomic Data

**Figure 8:**
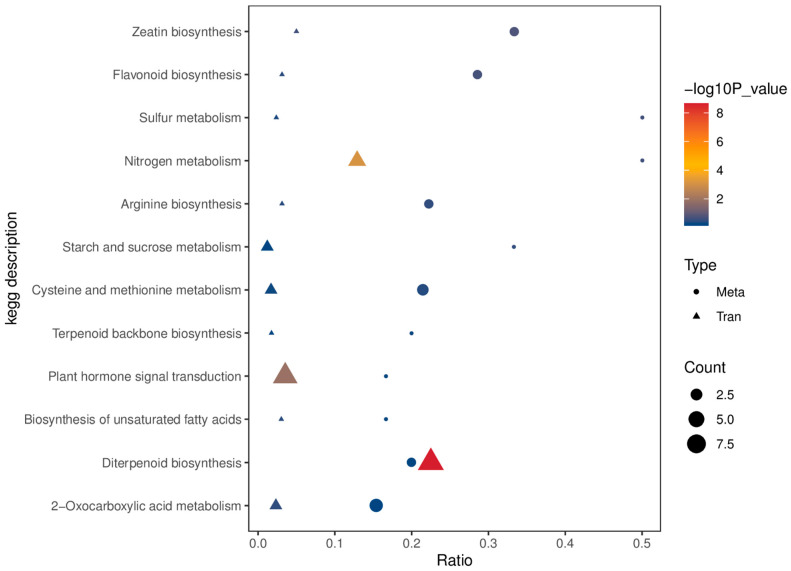
Correlation analysis of DEGs and DAMs between *cebip* vs. WT groups that were differentially expressed at 72 hpi.

The transcriptomic data showed that DEGs significantly expressed between the *cebip* and WT groups at 72 hpi are highly involved in CEBiP-mediated defense signaling pathways (Figure 4). To investigate if there is any relationship between DEGs and DAMs, we performed a correlation analysis for 439 DEGs and 421 DAMs, which were differentially expressed at 72 hpi. In total, 12 common enrichment pathways were identified between DEGs and DAMs, including zeatin biosynthesis, flavonoid biosynthesis, sulfur metabolism, nitrogen metabolism, arginine biosynthesis, starch and sucrose metabolism, cysteine and methionine metabolism, terpenoid backbone synthesis, plant hormone signal transduction, biosynthesis of unsaturated fatty acids, diterpenoid biosynthesis, and 2-Oxocarboxylic acid metabolism (Figure 8).

In total, 33 DEGs and 21 DAMs were correlated to 12 common enriched pathways. The specific DEGs and DAMs enriched in each category are shown in Table 2. Here, we looked into the details of three pathways: diterpenoid biosynthesis, nitrogen metabolism, and plant hormone signal transduction, which were identified as significantly enriched pathways for DEGs (*p* value < 0.05) (Table 2). However, DAMs enriched in these pathways did not show significance (*p* value > 0.05). In the nitrogen metabolism pathway, L-Glutamic acid was the only metabolite characterized and showed up-regulation between the *cebip* vs. WT groups at 72 hpi (Supplemental Table S2). Among four DEGs, NR1 showed up-regulation, and the others (alphaCA6, alphaCA4, and alphaCA3) exhibited down-regulation in the absence of CEBiP (Supplemental Table S3). Both diterpenoid biosynthesis and plant hormone signal transduction pathways included gibberellin family metabolites. Gibberellin A4 was detected in both pathways, while gibberellin A7 was solely found in the diterpenoid biosynthesis pathway (Table 2). Both gibberellin A4 and gibberellin A7 showed up-regulation between the *cebip* vs. WT groups at 72 hpi (Supplemental Table S2). The nine DEGs enriched in the diterpenoid biosynthesis pathway were all down-regulated between the *cebip* vs. WT groups at 72 hpi. In the plant hormone signal transduction category, PR1 family genes and MAPK5 were both down-regulated upon knockout of CEBiP, while the ethylene response sensor 2 (ERS2) gene showed up-regulation (Supplemental Table S3).

## 3. Discussion

In this study, we performed both RNA sequencing and LS-MS/MS analysis for rice leaves infected with *M. oryzae* by using the WT and the WT-derived CEBiP knockout (*cebip*) lines. Infected leaves were harvested at 24 hpi, 48 hpi, and 72 hpi and used for transcriptomic and metabolomic studies. Comparative analysis between the *cebip* vs. WT groups indicates that a total of 655 DEGs and 962 DAMs were significantly differentially expressed between the *cebip* vs. WT groups upon *M. oryzae* infection. KEGG analysis suggested DEGs were mainly associated with diterpenoid biosynthesis, phenylpropanoid biosynthesis, nitrogen metabolism, MAPK signaling pathway, plant–pathogen interaction, and plant hormone signal transduction. Knockout of CEBiP leads to the differential expression of a series of defense-related DEGs, including RLKs, PRs, WRKYs, and MYBs encoding genes. Metabolomic analysis reveals that DAMs showing differential expression were mainly involved in pathways like caffeine metabolism and glutathione metabolism. In the end, a correlation analysis reveals 12 common pathways enriched between DEGs and DAMs at 72 hpi. These data indicate potential signaling and metabolism pathways that were specifically involved in CEBiP-mediated resistance against *M. oryzae.*

RLPs rely on the intracellular domain of RLKs for downstream signal transduction [7,13]. Therefore, we suppose that knockout of CEBiP may lead to a differential expression of co-receptor RLKs. However, rice RLK CERK, which is known to be required for CEBiP-mediated immune response in rice cells [20], just showed a mild decrease in expression upon the knockout of CEBiP (Supplemental Figure S2). The CEBiP-CERK1 heterodimer has been well characterized in chitin signaling [20], but potential co-regulation of its gene expression during immune activation has not been experimentally demonstrated. In addition to CEBiP, RLPs LYP4 and LYP6 can also perceive chitin and activate a chitin-induced defense response in rice [26]. Similar to CEBiP, LYP4 and LYP6 form a complex with CERK upon chitin-induced defense signaling [27]. So far, whether there is crosstalk among CEBiP/CERK, LYP4/CERK, and LYP6/CERK signaling pathways is not clear. In the *cebip* line, it is possible that LYP4 or LYP6 somehow compensate the defense response in an alternative signaling pathway that operates independently of CEBiP. This may also explain the slight decrease in CERK expression in the *cebip* line upon *M. oryzae* infection.

Additionally, 29 RLK genes may contribute to the CEBiP-mediated defense response. These RLK genes present a significant down-regulation pattern in the *cebip* line compared to the WT line (Figure 5a). Among them, two genes encode RLCK family proteins, RLCK117 and RLCK62, which showed notable down-regulation at 24 hpi and 72 hpi, respectively, suggesting a positive role for them in the CEBiP-mediated defense response. This is in line with the findings that some RLCKs, like RLCK176, RLCK107, and RLCK185, positively contribute to chitin-induced immunity in a CERK-dependent manner [27,28,29]. Meanwhile, the other RLK families, including the LRR family, WAK family, L-Lec family, and so on (Figure 5a), also participate in the function of CEBiP. The crosstalk between diverse RLKs has been identified as a common mechanism in regulating plant immunity [13]. In Arabidopsis, RLK FLS2 recognizes the bacterial flg22 peptide and triggers BAK1-mediated phosphorylation of CERK1, thereby activating the downstream defense response [30]. Intriguingly, flg22-triggered phosphorylation of CERK1 by BAK1 establishes a primed conformation of CERK1 to further respond to fungal infections [30]. A similar case is also noticed for the Arabidopsis RLK NIK1, in which NIK1 and FLS2/BAK1 interplay in the immune response against viruses and bacteria, respectively [31]. It is possible that multiple RLKs co-regulate the immunity mediated by CEBiP, although the detailed regulation network needs further investigation.

Phytohormone salicylic acid (SA) seems to play a key role in the CEBiP-mediated defense response. SA is essential for the plant defense response against biotrophic pathogens, and activation of SA signaling leads to the up-regulation of the master regulator NPR1 and downstream PR proteins, like PR-1, PR-2, PR-5, and so on [32,33]. We found six PR1 family DEGs from the plant hormone signal transduction category showed significant down-regulation in the *cebip* line 72 hpi (Figure 5c), suggesting a positive role of the SA signaling pathway in the CEBiP-mediated defense response. Moreover, WRKY62, a transcriptional regulator previously demonstrated to function downstream of NPR1 in Arabidopsis [34], was also significantly down-regulated in the CEBiP knockout lines (Figure 5d). Unfortunately, we found that NH1 (the rice ortholog of NPR1) exhibited no significant differential expression (padj > 0.5; Supplemental Table S3), though it was still down-regulated in the *cebip* line at 72 hpi.

The other phytohormones, like abscisic acid (ABA), jasmonic acid (JA), ethylene (ET), and gibberellin (GA), may also participate in the CEBiP-mediated defense response. PP2C1 belongs to the PP2C family, and the PP2C family positively regulates ABA signaling in plants [35]. The up-regulation of PP2C1 in the CEBiP knockout line suggests a negative role of ABA in CEBiP function. Notably, the PP2C family gene AP2C1 negatively regulates MAPK6, thereby reducing JA/ET signaling in Arabidopsis against *Botrytis cinerea* [36]. This coincides with our findings on the contrary expression pattern between PP2C1 and MAPK6 in the CEBiP knockout lines (Supplemental Table S3). Elevated PP2C1 may suppress MAPK6 activation in CEBiP knockout lines, but this needs further experimental validation. MAPK6-derived MAPK cascades are essential for JA/ET signaling regulation in plants [37]. JA activation can induce the up-regulation of the PR10 family [38,39]. Our data show that the PR10 family gene JIOsPR10 showed reduced differential expression in the *cebip* lines (Supplemental Table S3), suggesting JA signaling was suppressed upon knockout of CEBiP. There might be crosstalk between ABA and JA signaling, in which the elevation of ABA signaling suppresses the JA signaling in CEBiP knockout lines. Surprisingly, an ethylene response sensor gene, ERS2, showed significant up-regulation in the absence of CEBiP (Supplemental Table S3). This conflicts with our speculation that PP2C1-mediated ABA signaling may also suppress MAPK6 downstream ET signaling. The observed up-regulation of ERS2 may not necessarily correlate with its functional activation, as ethylene receptor activity often requires proper conformational changes induced by ligand binding and subsequent phosphorylation events [40,41]. In correlation analysis between DEGs and DAMs, we found that Gibberellin A4 metabolite was identified in the plant hormone signal transduction category (Table 2), suggesting GA might also be involved in CEBiP immune function. Together, CEBiP employs diverse phytohormones to coordinate the plant defense response against *M. oryzae*, consistent with the interconnected phytohormone signaling networks acting as a conserved mechanism to fine-tune plant immune regulation [32,42,43].

Our metabolomic analysis suggests an important role of caffeine metabolism and glutathione metabolism in CEBiP-mediated signaling. Caffeine is a typical metabolite of purine alkaloids and is synthesized from xanthosine via three methylation steps [44]. Caffeine has been known to be toxic to various plant pathogens and herbivores, including bacteria, fungi, insects, and molluscs [45,46,47,48,49,50]. A recent study shows that caffeine can directly inhibit the growth of *M. oryzae*, and application of exogenous caffeine in rice enhances the resistance to *M. oryzae* [51]. Furthermore, the same study reveals that caffeine triggers the rice defense response via the Ca^2+^ signaling, which subsequently activates downstream SA signaling and expression of PR genes, including PR1 and PR10 [51]. Our data show that three metabolites (xanthosine, 7-Methyluric acid, and 5-Acetylamino-6-amino-3-methyluracil) enriched in caffeine metabolism all present a decreased accumulation in the CEBiP knockout lines (Supplemental Table S2). This coincides with the down-regulation of PR1 and PR10 in the transcriptomic data (Figure 5c). Glutathione is a non-protein thiol compound in plants and plays a key role in the defense response against multiple types of pathogens, like bacteria, fungi, and viruses [52,53,54]. During pathogen invasion, glutathione orchestrates redox homeostasis to regulate SA signaling pathways in an NPR1-dependent manner [53]. Within the glutathione metabolism category, we characterized three key metabolites (glutathione, L-glutamic acid, and Cys-Gly) that are essential for glutathione biosynthesis. Our data showed a time-dependent reduction in their accumulation levels that was markedly more severe in the *cebip* lines than in the WT lines (Supplemental Table S2). Combined with observed decreased SA signaling in transcriptomic data, it is possible that glutathione coordinates SA signaling to regulate CEBiP function. However, since glutathione also regulates JA/ET signaling, and sometimes even mediates the crosstalk between SA and JA/ET [53], how glutathione exactly participates in CEBiP downstream signaling remains to be further investigated.

## 4. Materials and Methods

### 4.1. Plant Materials

Rice cultivar Zhonghua 11 (ZH11) was used as the wild-type (WT) line in this study. For the *cebip* knockout mutant line, the mutation of the CEBiP gene in the ZH11 line was performed by Biogle Gene company via CRISPR-Cas9-mediated gene editing, as described online (http://www.biogle.cn/index/excrispr (accessed on 10 December 2021)). The mutation was confirmed via genomic DNA extraction, followed by sequencing using the specific primers CEBiP_CX_F: AACGTGATGCACCTCGCCTACAGCGT and CEBiP_CX_R: ATTAGCAAATGTTTATTATAGCATCAG. Both the WT and *cebip* lines were grown in the greenhouse at 25 °C under a 16-hour light/8-hour dark photoperiod.

### 4.2. Magnaporthe Oryzae Infection Assay

The *Magnaporthe oryzae* isolate Guy11 was grown in the incubator at 25 °C, 14 h light and 10 h dark photoperiod. Before use, the spore concentration of the Guy11 strain was adjusted to 1 × 10^5^ spores/mL with 0.5% gelatin. For the control group, leaves were sprayed with H_2_O with 0.5% gelatin. Two-week-old rice seedlings were spray-inoculated with adjusted conidia suspension and kept in a dark chamber at 22 °C for 48 h. Afterward, the plants were transferred to a growth chamber at 25 °C with 16 h light and 8 h dark. The disease symptoms were recorded by digital imaging at 5 days post infection. Thirty seedlings were used as a replicate for each treatment, and three technical replicates were used. For the transcriptome and metabolomics analyses, rice leaves were harvested at 24, 48, and 72 h post infection (hpi) and immediately frozen in liquid nitrogen.

### 4.3. RNA Extraction, Library Preparation, and Sequencing

Three biological replicates were collected for each treatment to perform transcriptome analysis. RNA extraction was performed using the RNAprep pure plant plus kit (Tiangen, Beijing, China), following the manufacturer’s protocol. RNA integrity and quantitation were assessed using the RNA Nano 6000 assay kit on a bioanalyzer 2100 system (Agilent Technologies, Santa Clara, CA, USA). The total RNA amount among samples ranges from 150 to app. 8750 ng, with the RIN value range from 4.90 to app. 8.0 (Supplementary Table S1). mRNA was first enriched from total RNA using poly-T oligo-attached magnetic beads. The purified mRNA was then subjected to first-strand cDNA synthesis using random hexamer primer and M-MuLV Reverse Transcriptase (RNase H-), followed by second-strand synthesis with DNA Polymerase I and RNase H. Final library preparation was performed with purification using the AMPure XP system. RNA sequencing was performed by Novogene (Beijing, China) using the Illumina Novaseq6000-PE150 platform (Illumina, San Diego, CA, USA), generating 150 bp paired-end reads for each sample.

### 4.4. Transcriptome Analysis

After quality control, the clean data were used for mapping to the *Oryza sativa* reference genome (ensembl_57_oryza_sativa_irgsp_1_0_toplevel) via Hisat2 v2.0.5. The Fragments Per Kilobase of transcript per Million mapped reads (FPKM) of each gene was calculated based on the length of the gene, and the reads count mapped to this gene. The principal component analysis (PCA) was conducted via the ggplot2 R package. Differential expression analysis was carried out using the DESeq2 R package (v1.20.0), based on the threshold of padj < 0.05 and log2 fold change > 2. The Venn diagram was performed via the VennDiagram R package (v3.5.0). To visualize the expression patterns of DEGs, the heatmap and K-means clustering were performed via ggplot2 and the pheatmap R package (v3.5.0). Gene Ontology (GO) and KEGG analysis were implemented by the clusterProfiler R package, and significant enrichment of the differentially expressed genes was characterized using a threshold setting of padj < 0.05.

### 4.5. Metabolite Extraction and MS Analysis

Six biological replicates were collected for each treatment for metabolomics analysis. Around 100 mg were ground with liquid nitrogen, and the homogenate was resuspended with precooled 80% methanol. The samples were chilled on ice for 5 min and followed by centrifugation at 15,000× *g*, 4 °C, for 20 min. Part of the supernatant was diluted by LC-MS grade water to a final concentration reaching 53% methanol. The samples were centrifuged at 15,000× *g*, 4 °C, for 20 min, and the supernatant was injected into the LC-MS/MS system for further analysis. The LC-MS/MS analysis was conducted by Novogene (Beijing, China) using a Vanquish UHPLC system (ThermoFisher, Bermen, Germany) coupled with an Orbitrap Q ExactiveTM HF mass spectrometer (ThermoFisher, Bermen, Germany). Samples went through the Hypersil Goldcolumn at a flow rate of 0.2 mL/min. The eluent A (0.1% FA in Water) and eluent B (Methanol) were used for the positive and negative polarity modes. The Q ExactiveTM HF mass spectrometer was operated in positive/negative polarity mode with the following conditions: spray voltage of 3.5 kV, capillary temperature of 320 °C, sheath gas flow rate of 35 psi, aux gas flow rate of 10 L/min, S-lens RF level of 60, and Aux gas heater temperature of 350 °C.

### 4.6. Data Process and Metabolomics Analysis

The raw data were processed using Compound Discoverer 3.3 (CD3.3, ThermoFisher) to implement peak alignment, peak picking, and quantitation for each metabolite. Peak intensities were normalized to the total spectral intensity. After that, data were predicted with the molecular formula according to the additive ions, molecular ion peaks, and fragment ions. The peaks were matched with the mzCloud (https://www.mzcloud.org/ (accessed on 17 December 2024)), mzVault, and MassList databases to obtain accurate qualitative and relative quantitative results. Statistical analyses were performed using R (v 3.4.3), Python (v 2.7.6), and CentOS (CentOS release 6.6). After quality control, the metabolites were annotated using the KEGG database (https://www.genome.jp/kegg/pathway.html (accessed on 14 Feburary 2025)). The metabolites with VIP > 1, *p*-value < 0.05, and log2 fold change > 1.5 were regarded as differentially accumulated metabolites. The Venn diagram was performed via the VennDiagram R package (v 3.5.0). In order to create the clustering heatmaps, the data were initially subjected to a process of normalization. This was accomplished by employing z-scores of the intensity areas of differential metabolites. The resulting data were then plotted using the Pheatmap package in R (v. 3.4.3). The functions of these metabolites and metabolic pathways were studied using the KEGG database.

## 5. Conclusions

**Figure 9:**
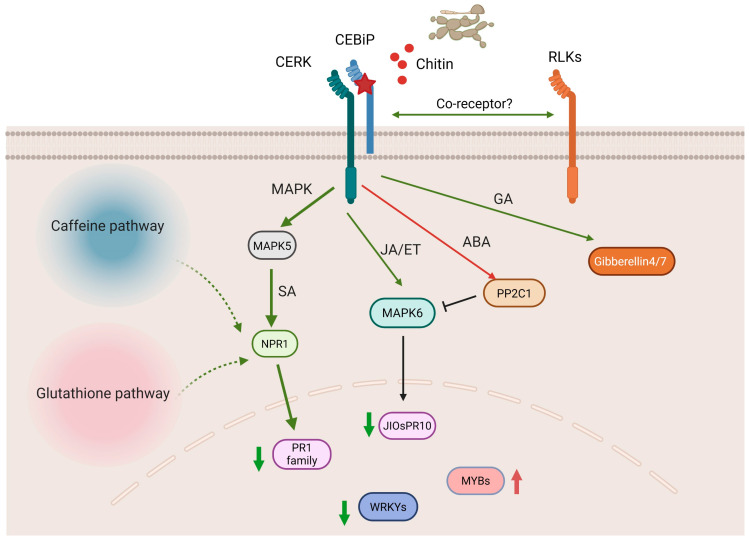
Overview of potential signaling and metabolism pathways involved in CEBiP-mediated resistance in rice against rice blast *Magnaporthe oryzae*. Upon the knockout of CEBiP, infection with *M. oryzae* leads to down-regulation of numerous RLK genes. Inside the cells, reduced MAPK signaling causes further down-regulation of SA response genes, like NPR1 and PR1 family genes. JA/ET/ABA/GA might be involved in the CEBiP-mediated defense response via the crosstalk with SA. Caffeine and glutathione metabolism might participate in CEBiP immune function through NPR1-dependent SA signaling. Red star, knockout of CEBiP. Red arrow, up-regulation. Green arrow, down-regulation. Dashed arrow, potential regulation.

In summary, in response to *M. oryzae* infection, the knockout of CEBiP leads to the down-regulation of a series of defense-related genes, including numerous RLK genes, PR genes, and TF genes. The RLKs genes might function as the co-receptors to contribute to the signaling activation inside the cells. The down-regulation of PR genes, especially PR1 family genes, suggests a positive role of SA signaling in the CEBiP-mediated defense response. Meanwhile, the other phytohormones, such as JA/ABA/ET/GA, may also be involved in CEBiP immune function. JA/ET might also contribute to CEBiP-mediated immune signaling, whereas ABA may negatively regulate CEBiP immune function. On the other hand, the metabolomic analysis suggests caffeine metabolism and glutathione metabolism are likely to be involved in the CEBiP-mediated defense response. Notably, caffeine and glutathione can participate in the SA signaling pathway during plant resistance. Through combined transcriptomic and metabolomic profiling, a comprehensive overview of CEBiP-mediated immune regulation emerges, with SA signaling identified as the dominant pathway driving the defense response (Figure 9). The data demonstrate that CEBiP relies on conserved immune signaling pathways to combat *M. oryzae*, and our findings further elucidate key metabolic pathways contributing to rice resistance.

## Figures and Tables

**Table 1:** KEGG pathways analysis reveals DEGs enriched in plant defense-related categories.

DEGs	Gene Name	Short Annotation	Plant–Pathogen Interaction (dosa04626)	MAPK Signaling Pathway (dosa04016)	Plant Hormone Signal Transduction (dosa04075)
Os03g0133400	CEBiP	Chitin elicitor-binding protein	✓		
Os01g0529800	Os01g0529800	3-ketoacyl-CoA synthase 6	✓		
Os07g0129200	PR1A	Pathogenesis-related protein PRB1–3	✓	✓	✓
Os07g0125500	Os07g0125500	Pathogenesis-related protein PR-1 type	✓	✓	✓
Os07g0124900	PR1#071	Pathogenesis-related protein PR-1 type	✓	✓	✓
Os07g0127500	PR1#072	Pathogenesis-related protein PRB1–2	✓	✓	✓
Os07g0127600	PR1#073	Pathogenesis-related protein 1	✓	✓	✓
Os02g0504700	Os02g0504700	-	✓		
Os06g0191500	Os06g0191500	Mitogen-activated protein kinase 5	✓	✓	✓
Os07g0129300	PR1	Pathogenesis-related protein 1	✓	✓	✓
Os05g0155200	ERS2	Probable ethylene response sensor 2		✓	✓
Os09g0325700	PP2C1	Probable protein phosphatase 2		✓	✓

**Table 2:** List of DEGs and DAMs in association analysis between *cebip* vs. WT groups at 72 hpi.

Pathway	DEGs	*p*-Value	DAMs	*p*-Value
Zeatin biosynthesis	Os10g0178500	0.2450	5′-Methylthioadenosine; O-beta-D-Glucosylzeatin	0.1746
Flavonoid biosynthesis	Os03g0819600	0.3625	Naringenin chalcone; Homoeriodictyol	0.2249
Nitrogen metabolism	Os08g0470200/Os08g0468100/ Os08g0423600/Os08g0423500	0.0008	L-Glutamic acid	0.2418
Sulfur metabolism	Os02g0167100	0.4465	O-Succinyl-L-homoserine	0.2418
Arginine biosynthesis	Os03g0279400	0.3625	L-Ornithine; L-Glutamic acid	0.3275
Starch and sucrose metabolism	Os01g0930800/Os02g0753000	0.6791	6-Phospho-beta-D-glucosyl-(1,4)-D-glucose	0.3402
Cysteine and methionine metabolism	Os05g0475400/Os02g0167100	0.4928	5′-Methylthioadenosine; Glutathione; O-Succinyl-L-homoserine	0.4050
Terpenoid backbone biosynthesis	Os07g0190000	0.5525	2-C-Methyl-D-erythritol 2,4-cyclodiphosphate	0.5009
Biosynthesis of unsaturated fatty acids	Os07g0417200	0.3714	alpha-Linolenic acid	0.5662
Plant hormone signal transduction	Os07g0127600/Os07g0127500/ Os07g0129300/Os07g0125500/ Os06g0191500/Os07g0124900/ Os07g0129200/Os05g0155200	0.0125	Gibberellin A4	0.5662
Diterpenoid biosynthesis	Os11g0474800/Os04g0179200/ Os12g0491800/Os04g0178300/ Os04g0179700/Os04g0178400/ Os02g0570400/Os02g0569900/ Os06g0568600	2.22E-09	Gibberellin A4; Gibberellin A7	0.6244
2-Oxocarboxylic acid metabolism	Os05g0475400/Os03g0279400	0.3376	L-Ornithine; 3-(5′-Methylthio)pentylmalic acid; cis-(Homo)3-aconitate; Citraconic acid	0.7603

## Data Availability

The raw data of the transcriptome have been uploaded to NCBI (project number: PRJNA1250220), and other data that support the findings of this study are available on request from the corresponding author.

## Supplementary Materials

The following supporting information can be downloaded at: https://www.mdpi.com/article/10.3390/ijms26115194/s1.
