# Supplementary figures and images for: Integrated Transcriptomic and Metabolomic Analysis Reveals the Regulation Network of CEBiP in Rice Defense Against Magnaporthe oryzae
Source: Int J Mol Sci. 2025 May 28;26(11):5194. doi: 10.3390/ijms26115194 (PMC12154221; doi:10.3390/ijms26115194)

## Supplementary Materials

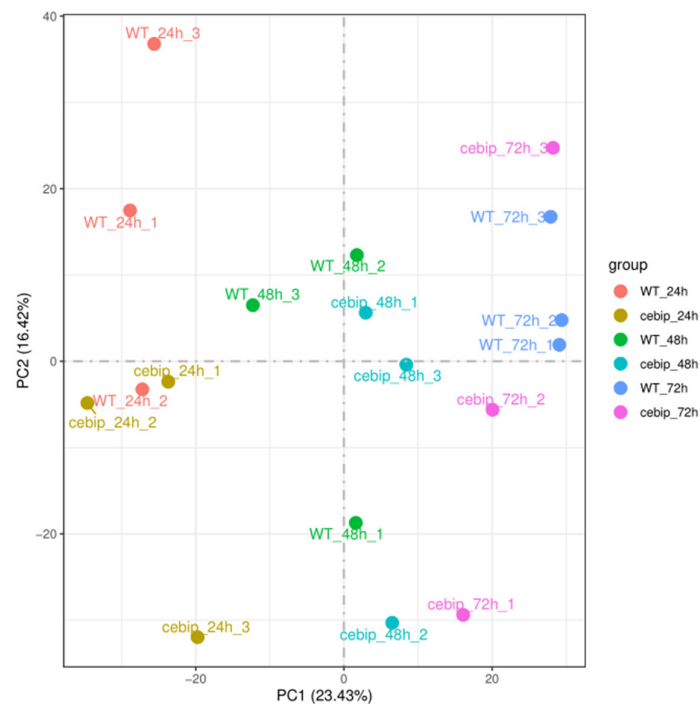

Supplement: Supplementary file 1 [file ijms-26-05194-s001.zip › Supplementary materials.pdf]
